# COL0RME: Super-resolution microscopy based on sparse blinking/fluctuating fluorophore localization and intensity estimation

**DOI:** 10.1017/S2633903X22000010

**Published:** 2022-02-16

**Authors:** Vasiliki Stergiopoulou, Luca Calatroni, Henrique de Morais Goulart, Sébastien Schaub, Laure Blanc-Féraud

**Affiliations:** 1 CNRS, INRIA, I3S, Université Côte d’Azur, Sophia Antipolis, France; 2 IRIT, Université de Toulouse, CNRS, Toulouse INP, Toulouse, France; 3 CNRS, LBDV, Sorbonne Université, Villefranche-sur-Mer, France

**Keywords:** Fluorescence microscopy, SOFI method, sparse optimization, super-resolution

## Abstract

To overcome the physical barriers caused by light diffraction, super-resolution techniques are often applied in fluorescence microscopy. State-of-the-art approaches require specific and often demanding acquisition conditions to achieve adequate levels of both spatial and temporal resolution. Analyzing the stochastic fluctuations of the fluorescent molecules provides a solution to the aforementioned limitations, as sufficiently high spatio-temporal resolution for live-cell imaging can be achieved using common microscopes and conventional fluorescent dyes. Based on this idea, we present COL0RME, a method for covariance-based 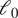 super-resolution microscopy with intensity estimation, which achieves good spatio-temporal resolution by solving a sparse optimization problem in the covariance domain and discuss automatic parameter selection strategies. The method is composed of two steps: the former where both the emitters’ independence and the sparse distribution of the fluorescent molecules are exploited to provide an accurate localization; the latter where real intensity values are estimated given the computed support. The paper is furnished with several numerical results both on synthetic and real fluorescence microscopy images and several comparisons with state-of-the art approaches are provided. Our results show that COL0RME outperforms competing methods exploiting analogously temporal fluctuations; in particular, it achieves better localization, reduces background artifacts, and avoids fine parameter tuning.

## Impact Statement

This research paper describes a super-resolution method improving the spatial resolution of images acquired by common fluorescence microscopes and conventional blinking/fluctuating fluorophores. The problem is formulated in terms of a sparse and convex/nonconvex optimization problem in the covariance domain for which a well-detailed algorithmic and numerical description are provided. It is addressed to an audience working at the interface between applied mathematics and biological image analysis. The proposed approach is validated on several synthetic datasets and shows promising results also when applied to real data, thus paving the way for new future research directions.

## 1. Introduction

In the field of fluorescence (or, more generally, light) microscopy, the main factor characterizing the microscope resolution is the limit imposed by the diffraction of light: structures with size smaller than the diffraction barrier (typically around 250 nm in the lateral direction) cannot be well distinguished nor localized. The need to investigate small subcellular entities thus led to the implementation of a plethora of super-resolution methods.

A large and powerful family of imaging techniques achieving nanometric resolution are the ones often known as single molecule localization microscopy (SMLM) techniques, see, for example, Refs. (1) and (2) for a review. Among them, methods such as photo-activated localization microscopy (PALM)^(^^3^^)^ and stochastic optical reconstruction microscopy (STORM)^(^^4^^)^ are designed so as to create a super-resolved image (achieving around 20 nm of resolution) by activating and precisely localizing only a few molecules in each of thousands of acquired frames at a time. For their use, these methods need specific photoactivatable, photoswitchable, and binding-activated fluorophores, among others^(^^5^^)^, as well as, a large number (typically thousands) of sparse acquired frames leading to a poor temporal resolution and large exposure times which can significantly damage the sample. A different technique improving spatial resolution is well-known under the name of stimulated emission depletion (STED) microscopy^(^^6^^)^. Similarly to SMLM, STED techniques are based on a time-consuming and possibly harmful acquisition procedure requiring special equipment. In STED microscopy, the size of the point spread function (PSF) is reduced as a depletion beam of light will induce stimulated emission from molecules outside the region of interest and thus switch them off. Structured illumination microscopy (SIM)^(^^7^^)^ methods use patterned illumination to excite the sample; differently from the aforementioned approaches, images here can be recovered with high temporal-resolution via high-speed acquisitions that cause comparatively little damage to the sample, but at the cost of a relatively low spatial resolution and, more importantly, the requirement of a specific illumination setup. Note that in this paper, we address grid-based super-resolution approaches, that is, the ones that formalize the super-resolution problem as the task of retrieving a well-detailed image on a fine grid from coarse measurements. More recently, off-the-grid super-resolution approaches have started to be studied in the literature, such as the one of Candès *et al.*^(^^8^^)^, with applications to SMLM data in Denoyelle *et al.*^(^^9^^)^, as well as DAOSTORM^(^^10^^)^, a high-density super-resolution microscopy algorithm. The great advantage of the gridless approaches is that there are no limitations imposed by the size of the discrete grid considered. However, both the theoretical study of the problem and its numerical realization become very hard due to the infinite-dimensional and typically nonconvex nature of the optimization.

During the last decade, a new approach taking advantage of the independent stochastic temporal fluctuations/blinking of conventional fluorescent emitters appeared in the literature. A stack of images is acquired at a high temporal rate, typically 20–100 images/s, by means of common microscopes (such as widefield, confocal, or total internal reflection fluorescence [TIRF] ones) using standard fluorophores, and then their independent fluctuations/blinking are exploited so as to compute a super-resolved image. Note that no specific material is needed here, neither for the illumination setup nor for fluorophores. Several methods exploiting the sequence of images have been proposed over the last years. Due to standard acquisition settings, temporal resolution properties are drastically improved. To start with, super-resolution optical fluctuation imaging (SOFI)^(^^11^^)^ is a powerful technique where second and/or higher-order statistical analysis is performed, leading to a significant reduction of the size of the PSF. An extension of SOFI that combines several cumulant orders and achieves better resolution levels than SOFI is the method bSOFI^(^^12^^)^. However, spatial resolution still cannot reach the same levels of PALM/STORM. Almost the same behavior has been noticed in super-resolution radial fluctuations (SRRF)^(^^13^^)^ microscopy, where super-resolution is achieved by calculating the degree of local symmetry at each frame. Despite its easy manipulation and broad applicability, SRRF creates significant reconstruction artifacts which may limit its use in view of accurate analysis. Other methods which belong to the same category and are worth mentioning are: the method 3B^(^^14^^)^, which uses Bayesian analysis and takes advantage of the blinking and bleaching events of standard fluorescent molecules, the method entropy-based super-resolution imaging (ESI)^(^^15^^)^ that computes entropy values pixel-by-pixel, weighted with higher-order statistics and the method spatial covariance reconstructive (SCORE)^(^^16^^)^ that analyzes intensity statistics, similarly to SOFI, but further reduces noise and computational cost by computing only a few components that have a significant contribution to the intensity variances of the pixels. In addition, the approach sparsity-based super-resolution correlation microscopy (SPARCOM)^(^^17^^,^^18^^)^ exploits, as SOFI, both the lack of correlation between distinct emitters as well as the sparse distribution of the fluorescent molecules via the use of an 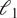 regularization defined on the emitters’ covariance matrix. Along the same lines, a deep-learning method exploiting algorithmic unfolding, called learned SPARCOM (LSPARCOM)^(^^19^^)^, has recently been introduced. Differently from plain SPARCOM, the advantage of LSPARCOM is that neither previous knowledge of the PSF nor any heuristic choice of the regularization parameter for tuning the sparsity level is required. As far as the reconstruction quality is concerned, both SPARCOM and LSPARCOM create some artifacts under challenging imaging conditions, for example, when the noise and/or background level are relatively high. Finally, without using higher-order statistics, a constrained tensor modeling approach that estimates a map of local molecule densities and their overall intensities, as well as, a matrix-based formulation that promotes structure sparsity via an 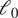-type regularizer, are available in Ref. (20). These approaches can achieve excellent temporal resolution levels, but the spatial resolution is limited.

### 1.1. Contribution

**Figure 1.**
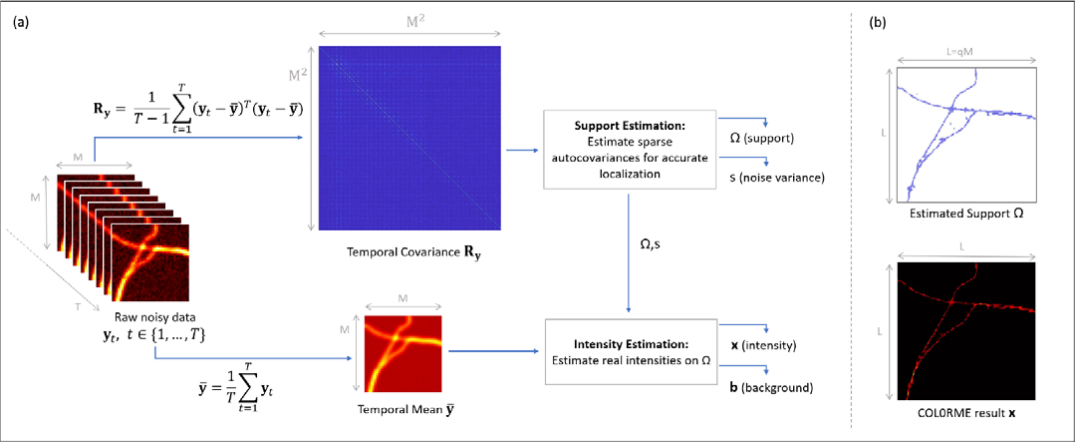
Principles of COL0RME. (a) An overview of the two steps (support estimation and intensity estimation) by visualizing the inputs/outputs of each, as well as the interaction between them. (b) The two main outputs of COL0RME are: the support 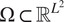 containing the locations of the fine-grid pixels with at least one fluorescent molecule, and the intensity 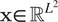 whose non-null values are estimated only on 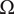.

In this paper, we propose a method for live-cell super-resolution imaging based on the sparse analysis of the stochastic fluctuations of molecule intensities. The proposed approach provides a good level of both temporal and spatial resolution, thus allowing for both precise molecule localization and intensity estimation at the same time, while relaxing the need for special equipment (microscope, fluorescent dyes) typically encountered in state-of-the-art super-resolution methods such as, for example, SMLM. The proposed method is called COL0RME, which stands for covariance-based super-resolution microscopy with intensity estimation.The principles of COL0RME are shown in Figure 1. Similarly to SPARCOM^(^^18^^)^, COL0RME enforces signal sparsity in the covariance domain by means of sparsity-promoting terms, either of convex (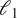, TV) or nonconvex (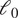-based)-type. Differently from SPARCOM, COL0RME allows also for an accurate estimation of the noise variance in the data and is complemented with an automatic selection strategy of the model hyperparameters. Furthermore, and more importantly, COL0RME allows for the estimation of both signal and background intensity, which are relevant pieces of information for biological studies. By exploiting information on the estimated noise statistics, the parameter selection in this step is also made fully automatic, based on the standard discrepancy principle. We remark that an earlier version of COL0RME has been already introduced by the authors in Ref. (21). Here, we consider an extended formulation combined with automatic parameter selection strategies which allows for the analysis of more challenging data having, for example, spatially varying background. The method is validated on simulated and tested on challenging real data. Our results show that COL0RME outperforms competing methods in terms of localization precision, parameter tuning and removal of background artifacts.

## 2. Mathematical Modeling

For real scalars 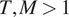 and 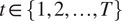, let 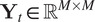 be the blurred, noisy, and down-sampled image frame acquired at time 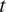. We look for a high-resolution image 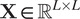 being defined as 
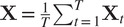
 with 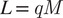 and defined on a 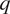-times finer grid, with 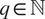. Note that in the following applications, we typically set 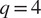. The image formation model describing the acquisition process at each 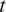 can be written as:(1)
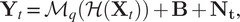
where 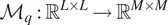 is a down-sampling operator summing every 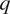 consecutive pixels in both dimensions, 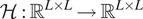 is a convolution operator defined by the PSF of the optical imaging system and 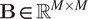 models the background, which collects the contributions of the out-of-focus (and the ambient) fluorescent molecules. Motivated by experimental observations showing that the blinking/fluctuating behavior of the out-of-focus molecules is not visible after convolution with wide de-focused PSFs, we assume that the background is temporally constant (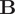 does not depend on 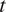), while we allow it to smoothly vary in space. Finally, 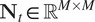 describes the presence of noise modeled here as a matrix of independent and identically distributed (i.i.d.) Gaussian random variables with zero mean and variance 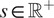 taking into account both the underlying electronic noise and the noise bias induced by 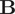 (see Remark 1 for more details on the approximation considered). We assume that the molecules are located at the center of each pixel and that there is no displacement of the specimen during the imaging period, which is a reasonable assumption whenever short time acquisitions are considered.

**Remark 1.** *A more appropriate model taking also into account the presence of signal-dependent Poisson noise in the data would be the following:*(2)
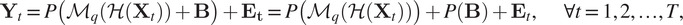
*where, for* 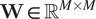, 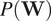 *represents the realization of a multivariate Poisson variable of parameter* 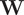 *and* 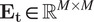 *models electronic noise with a matrix of i.i.d. Gaussian entries of zero mean and constant variance* 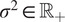
*. Note that the second equality in* (2) *holds due to the independence between* 
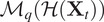
 *and* 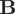
*. Model* (2) *is indeed the one we used for the generation of the simulated data, see Section 6.1. However, to simplify the reconstruction process, we simplified* (2) *by assuming that* 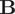 *has sufficiently large entries, so that* 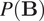 *can be approximated as* 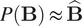 *with* 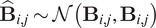
*, where* 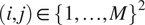
*, thus considering:*(3)
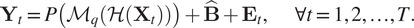
*By now further approximating the variance of* 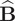 *with a constant* 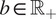 *to be interpreted as the average of* 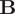
*, we have that by simple manipulations:*
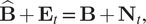
*where the independence between* 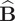 *and* 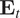 *has been exploited. We can thus retrieve* (1) *from* (3) *by neglecting the Poisson noise dependence in* 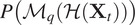 *and that the variance of every entry of the random term* 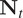 *is* 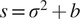. *A more detailed and less approximated modeling taking into account the signal-dependent nature of the noise in the data could represent a very interesting area of future research.*

In vectorized form, model (1) reads:(4)
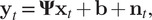
where 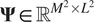 is the matrix representing the composition 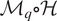, while 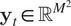, 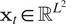, 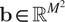, and 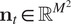 are the column-wise vectorizations of 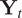, 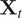, 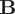, and 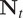 in (1), respectively.

For all 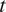 and given 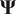 and 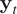, the problem can thus be formulated as
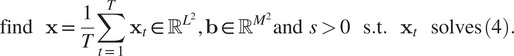
In order to exploit the statistical behavior of the fluorescent emitters, we reformulate the model in the covariance domain. This idea was previously exploited by the SOFI approach^(^^11^^)^ and was shown to significantly reduce the full-width-at-half-maximum (FWHM) of the PSF. In particular, the use of second-order statistics for a Gaussian PSF corresponds to a reduction factor of the FWHM of 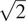.

To formulate the model, we consider the frames 
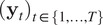
 as 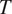 realizations of a random variable 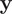 with covariance matrix defined by:(5)
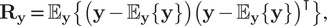
where 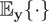 denotes the expected value computed w.r.t. to the unknown law of 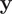. We estimate 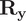 by computing the empirical covariance matrix, that is,
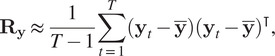
where 
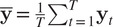
 denotes the empirical temporal mean. From (4) and (5), we thus deduce the relation:(6)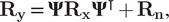 where 
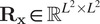
 and 
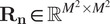
 are the covariance matrices of 
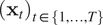
 and 
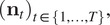
 respectively. As the background is stationary by assumption, the covariance matrix of 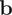 is zero. Recalling now that the emitters are uncorrelated by assumption, we deduce that 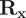 is diagonal. We thus set 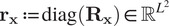. Furthermore, by the i.i.d. assumption on 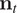, we have that 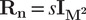, where 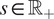 and 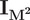 is the identity matrix in 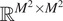. Note that the model in equation (6) is similar to the SPARCOM one presented in Ref. (18), with the difference that here we consider also noise contributions by including in the model the diagonal covariance matrix 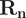. Finally, the vectorized form of the model in the covariance domain can thus be written as:
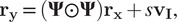
where 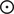 denotes the Khatri–Rao (column-wise Kronecker) product, 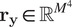 is the column-wise vectorization of 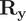 and 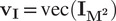.

## 3. COL0RME, Step I: Support Estimation for Precise Molecule Localization

Similarly to SPARCOM^(^^18^^)^, our approach makes use of the fact that the solution 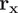 is sparse, while including further the estimation of 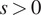 for dealing with more challenging scenarios. In order to compare specific regularity *a priori* constraints on the solution, we make use of different regularization terms, whose importance is controlled by a regularization hyperparameter 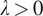. By further introducing some non-negativity constraints for both variables 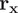 and 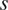, we thus aim to solve:(7)
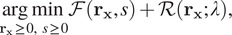
where the data fidelity term is defined by:(8)
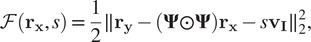
and 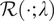 is a sparsity-promoting penalty. Ideally, one would like to make use of the 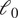 norm to enforce sparsity. However, as it is well-known, solving the resulting noncontinuous, nonconvex, and combinatorial minimization problem is an NP-hard problem. A way to circumvent this difficulty consists in using the continuous exact relaxation of the 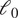 norm (CEL0) proposed by Soubies *et al.* in Ref. (22). The CEL0 regularization is continuous, nonconvex and preserves the global minima of the original 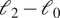 problem while removing some local ones. It is defined as follows:(9)
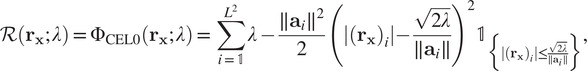
where 
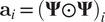
 denotes the 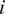
th column of the operator 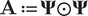.

A different, convex way of favoring sparsity consists in taking as regularizer the 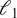 norm, that is,(10)
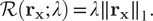
Besides convexity and as it is well-known, the key difference between using the 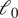 and the 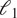-norm is that the 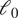 provides a correct interpretation of sparsity by counting only the number of the nonzero coefficients, while the 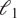 depends also on the magnitude of the coefficients. However, its use as a sparsity-promoting regularizer is nowadays well-established (see, e.g., Ref. (23)) and also used effectively in other microscopy applications, such as SPARCOM^(^^18^^)^.

**Figure 2.**
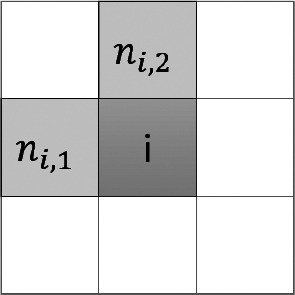
The one-sided nearest horizontal and vertical neighbors of the pixel 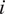 used to compute the gradient discretization in (11).

Finally, in order to model situations where piece-wise constant structures are considered, we consider a different regularization term favoring gradient-sparsity using the total variation (TV) regularization defined in a discrete setting as follows:(11)
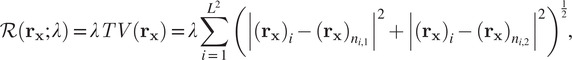
where 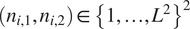 indicate the locations of the horizontal and vertical nearest neighbor pixels of pixel 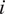, as shown in Figure 2. For the computation of the TV penalty, Neumann boundary conditions have been used.

To solve (7), we use the alternate minimization algorithm between 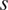 and 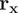
^(^^24^^)^, see the pseudo-code reported in Algorithm 1. Note that, at each 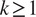, the update for the variable 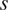 can be efficiently computed through the following explicit expression:
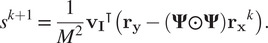


Concerning the update of 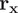, different algorithms were used depending on the choice of the regularization term in (9)–(11). For the CEL0 penalty (9), we used the iteratively reweighted 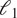 algorithm (IRL1)^(^^25^^)^, following Gazagnes *et al.*^(^^26^^)^ with fast iterative shrinkage-thresholding algorithm (FISTA)^(^^27^^)^ as inner solver. If the 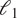 norm (10) is chosen, FISTA is used. Finally, when the TV penalty (11) is employed, the primal-dual splitting method in Ref. (28) was considered.

Algorithm 1 **COL0RME, Step I: Support Estimation**

**Require**: 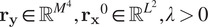.

**repeat**

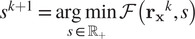

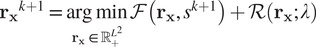

**until** convergence.

**return** 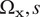.

Following the description provided by Attouch *et al.* in Ref. (24), convergence of Algorithm 1 can be guaranteed only if an additional quadratic term is introduced in the objective function of the second minimization subproblem. Nonetheless, empirical convergence was observed also without such additional terms.

**Figure 3.**
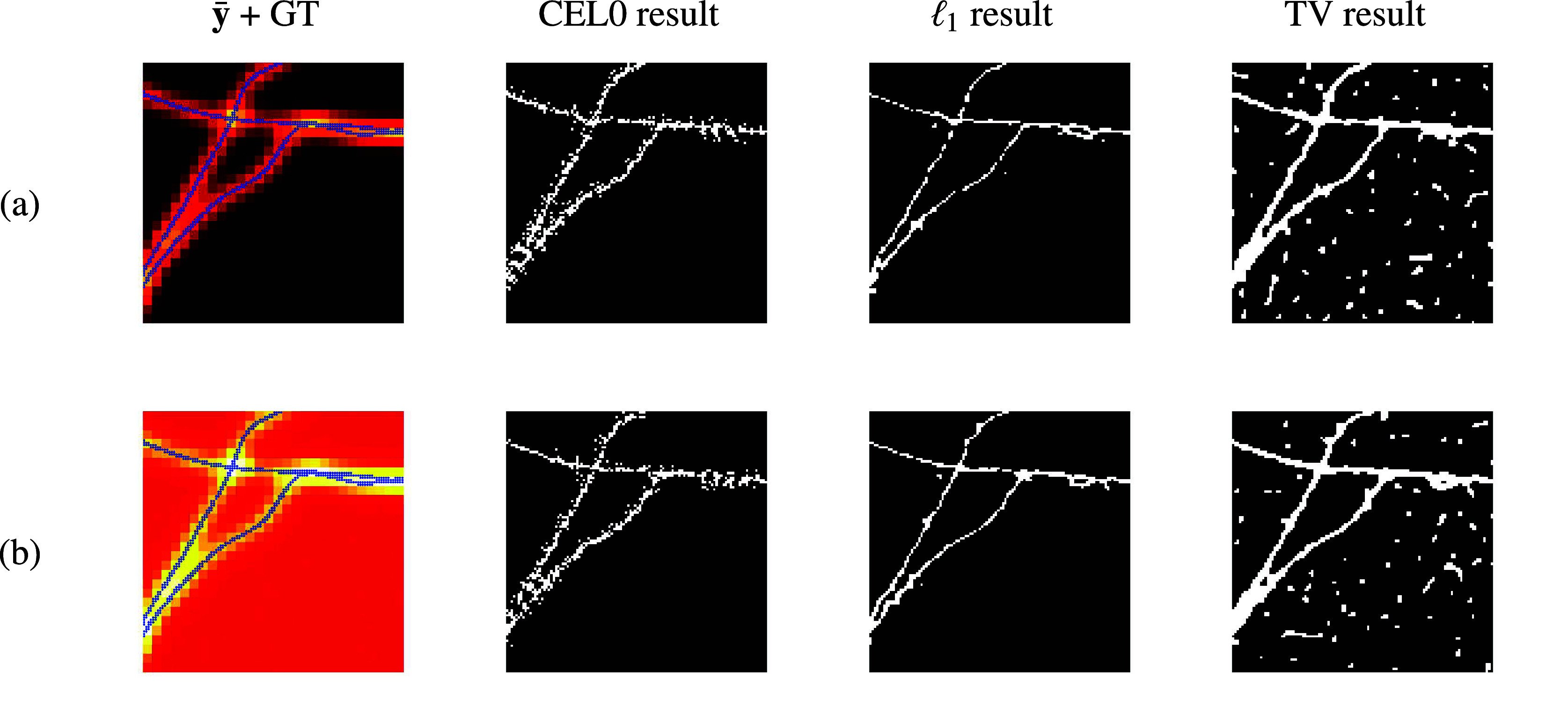
(a) Noisy simulated dataset with low background (LB) and stack size: 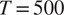 frames, (b) Noisy simulated high-background (HB) dataset, with 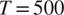 frames. From left to right: Superimposed diffraction limited image (temporal mean of the stack) with 4× zoom on ground truth support (blue), CEL0 reconstruction, 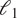 reconstruction and total variation (TV) reconstruction.

To evaluate the performance of the first step of the method COL0RME using the different regularization penalties described above, we created two noisy simulated datasets, with low background (LB) and high background (HB), respectively and used them to apply COL0RME and estimate the desired sample support. More details on the two datasets are available in the following Section 6.1. The results obtained using the three different regularizers are reported in Figure 3. In this example, we chose the regularization parameter 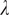 heuristically, while more details about the selection of the parameter are given in the Section 5.1.

Despite its continuous and smooth reconstruction, we observe that the reconstruction obtained by the TV regularizer does not provide precise localization results. For example, the separation of the two filaments on the top-right corner is not visible and while the junction of the other two filaments on the bottom-left should appear further down, we clearly see that those filaments are erroneously glued together. Nonetheless, the choice of an appropriate regularizer tailored to favor fine structures as the ones observed in the GT image constitutes a challenging problem that should be addressed in future research.

The Jaccard indices (JIs) of both the results obtained when using the CEL0 and 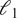 regularizer, that allow for more precise localization, have been computed. The JI is a quantity in the range 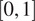 computed as the ratio between correct detections (CD) and the sum of correct detections, false positives (FP) and false negatives (FN), that is, 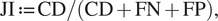 up to a tolerance 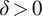, measure in nm. A correct detection occurs when one pixel at most 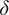 nm away from a ground truth pixel is added to the support. In order to match the pixels from the estimated support to the ones from the ground truth, we employ the standard Gale–Shapley algorithm^(^^29^^)^. Once the matching has been performed, we can simply count the number of ground truth pixels which have not been detected (false negatives) and also the number of pixels in the estimated support which have not been matched to any ground truth pixel (false positives).

**Figure 4.**
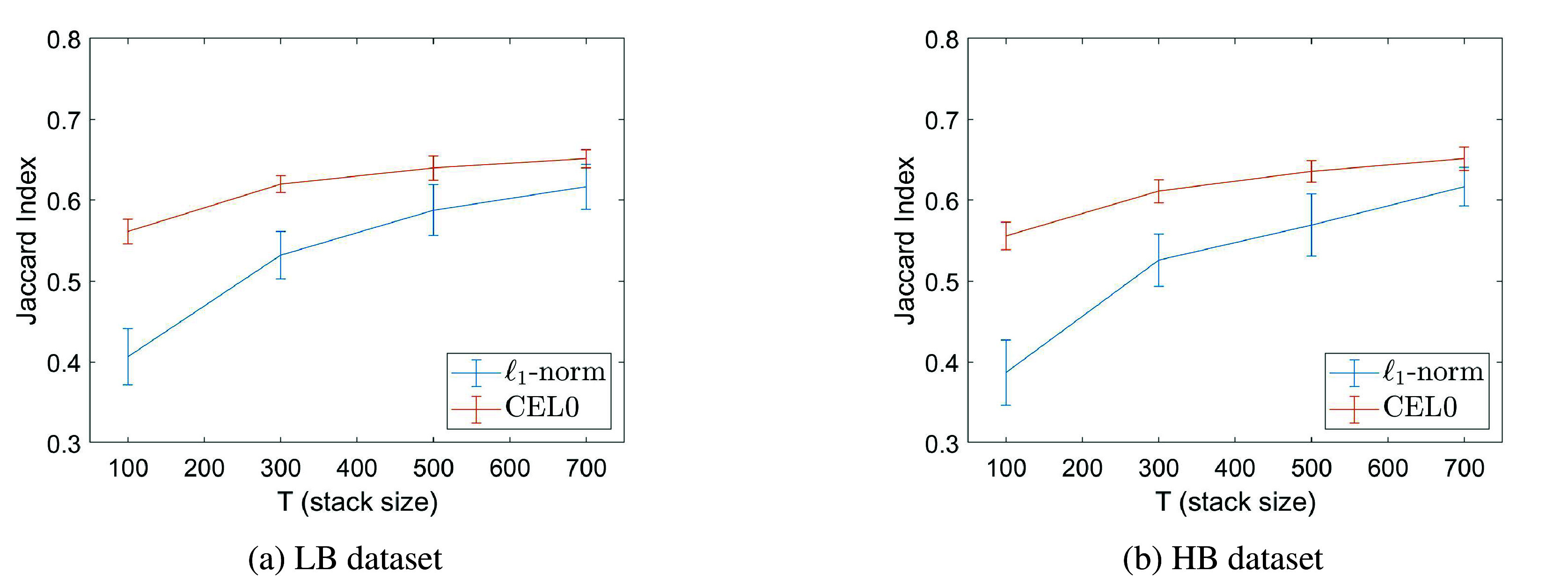
Jaccard Index values with tolerance 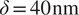 for the low-background (LB) and high-background (HB) datasets, for different stack sizes and regularization penalty choices. The tolerance, 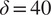 nm, is set so that we allow the correct detections, that needed to be counted for the computation of the Jaccard Index, to be found not only in the same pixel but also to any of the 8-neighboring pixels.

Figure 4 reports the average JI computed from 20 different noise realizations, as well as, an error bar (vertical lines) that represent the standard deviation, for several stack sizes. According to the figure, a slightly better JI is obtained when the CEL0 regularizer is being used, while an increase in the number of frames, when both regularizers being used, leads to better JI, hence better localization. As the reader may notice, such quantitative assessment could look inconsistent with the visual results reported in Figure 3. By definition, the JI tends to assume higher values whenever more CD are found even in presence of more FP (as it happens for the CEL0 reconstruction), while it gets more penalized when FN happen, as they affect the computation “twice,” reducing the numerator and increasing the denominator.

### 3.1. Accurate noise variance estimation

**Figure 5.**
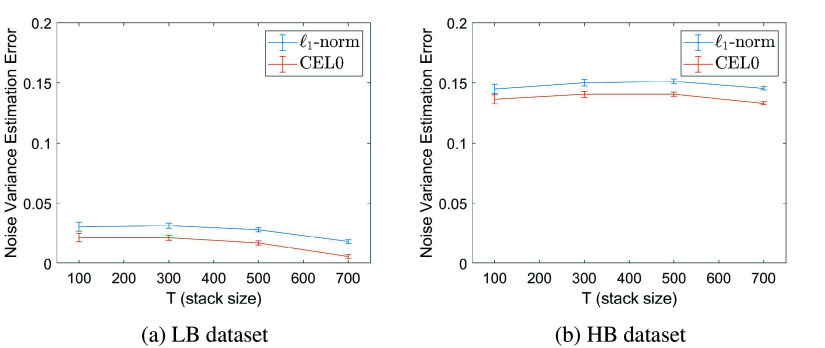
The relative error in noise variance estimation, defined as: Error = 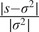, where 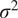 is the constant variance of the electronic noise. The error is computed for 20 different noise realizations, presenting in the graph the mean and the standard deviation (error bars).

Along with the estimations of the emitter’s temporal sparse covariance matrix, the estimation of the noise variance in the joint model (7) allows for much more precise results even in challenging acquisition conditions. In Figure 5, we show the relative error between the computed noise variance 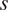 and the constant variance of the electronic noise 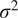 used to produce simulated LB and HB data. The relative error is higher in the case of the HB dataset, something that is, expected, as in our noise variance estimation 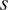 there is a bias coming from the background (see Remark 1). In the case of the LB dataset, as the background is low, the bias is sufficiently small so that it is barely visible in the error graph. In our experiments, a Gaussian noise with a corresponding SNR of approximately 16 dB is being used, while the value of 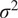 is in average equal to 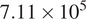 for the LB dataset and 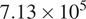 for the HB dataset. Note that, in general, the estimation of the noise variance 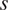 obtained by COL0RME is very precise.

## 4. COL0RME, Step II: Intensity Estimation

From the previous step, we obtain a sparse estimation of 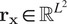. Its support, that is, the location of nonzero variances, can thus be deduced. This is denoted in the following by 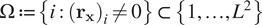. Note that this set corresponds indeed to the support of the desired 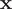, hence in the following we will use the same notation to denote both sets.

We are now interested in enriching COL0RME with an additional step where intensity information of the signal 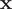 can be retrieved in correspondence with the estimated support 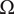. To do so, we thus propose an intensity estimation procedure for 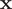 restricted only to the pixels of interest. Under this modeling assumption, it is thus reasonable to consider a regularization term favoring smooth intensities on 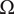, in agreement to the intensity typically found in real images.

In order to take into account the modeling of blurry and out-of-focus fluorescent molecules, we further include in our model (4) a regularization term for smooth background estimation. We can thus consider the following joint minimization problem:(12)
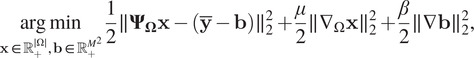
where the data term models the presence of Gaussian noise, 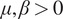 are regularization parameters and the operator 
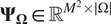
 is a matrix whose 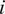
th column is extracted from 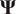 for all indices 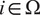. Finally, the regularization term on 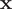 is the squared norm of the discrete gradient restricted to 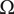, that is,
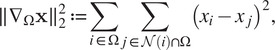
where 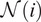 denotes the 8-pixel neighborhood of 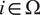. Note that, according to this definition, 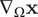 denotes a (redundant) isotropic discretization of the gradient of 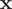 evaluated for each pixel in the support 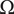. Note that this definition coincides with the standard one for 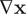 restricted to points in the support 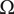.

The non-negativity constraints on 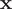 and 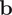 as well as the one restricting the estimation of 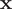 on 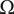 can be relaxed using suitable smooth penalty terms, so that, finally, the following optimization problem can be addressed:(13)
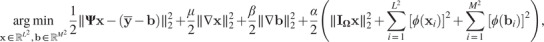
where the parameter 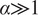 can be chosen arbitrarily high to enforce the constraints, 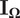 is a diagonal matrix acting as characteristic function of 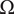, that is, defined as:
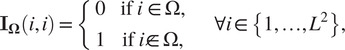
and 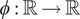 is used to penalize negative entries, being defined as:(14)
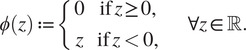
We anticipate here that considering the unconstrained problem (13) instead of the original, constrained, one (12), will come in handy for the design of an automatic parameter selection strategy, as we further detail in Section 5.2.

To solve the joint-minimization problem (13), we use the Alternate Minimization algorithm, see Algorithm 2. In the following subsections, we provide more details on the solution of the two minimization subproblems.

Algorithm 2 **COL0RME, Step II: Intensity Estimation.**

**Require:** 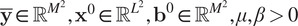, 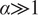.

**repeat**

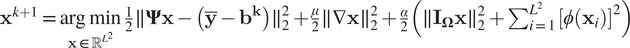

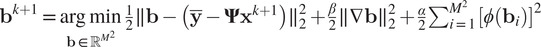

**until** convergence.

**return** 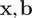.

### 4.1. First subproblem: update of *x*

In order to find at each 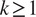 the optimal solution 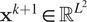 for the first subproblem, we need to solve a minimization problem of the form:(15)
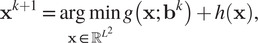
where, for 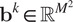 being fixed at each iteration 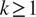, 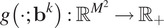 is a proper and convex function with Lipschitz gradient, defined as:(16)
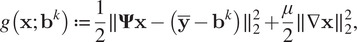
and where the function 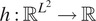 encodes the penalty terms:(17)
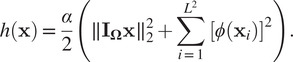


Solution of (15) can be obtained iteratively, using, for instance, the proximal gradient descent algorithm, whose iteration can be defined as follows:(18)
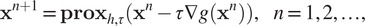
where 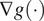 denotes the gradient of 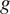, 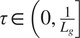 is the algorithmic step-size chosen inside a range depending on the Lipschitz constant of 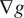, here denoted by 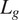, to guarantee convergence. The proximal update in (18) can be computed explicitly using the computations reported in Appendix A. One can show in fact that, for each 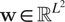 there holds element-wise:(19)
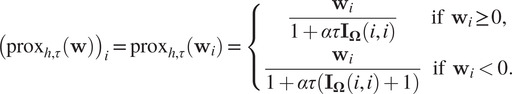

**Remark 2.** *As the reader may have noted, we consider the proximal gradient descent algorithm* (18) *for solving* (15)*, even though both functions* 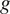 *and* 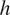 *in* (16) *and* (17) *respectively, are smooth and convex, hence, in principle, (accelerated) gradient descent algorithms could be used. Note, however, that the presence of the large penalty parameter* 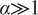 *would significantly slow down convergence speed in such case as the step size* 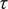 *in this case would be constrained to the smaller range* 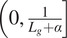
*. By considering the penalty contributions in terms of their proximal operators, this limitation does not affect the range of* 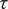 *and convergence is still guaranteed^(^**^30^**^)^ in a computationally fast way through the update* (19).

### 4.2. Second subproblem: update of *b*

As far as the estimation of the background is concerned, the minimization problem we aim to solve at each 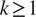 takes the form:(20)
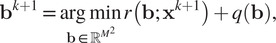
where
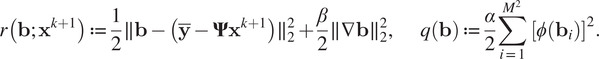
Note that 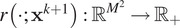 is a convex function with 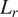-Lipschitz gradient and 
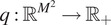
 encodes (large, depending on 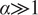) penalty contributions. Recalling Remark 2, we thus use again the proximal gradient descent algorithm for solving (20). The desired solution 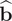 at each 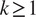 can thus be found by iterating:(21)
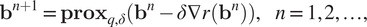
for 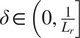. The proximal operator 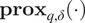, has an explicit expression and it is defined element-wise for 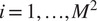 as:(22)
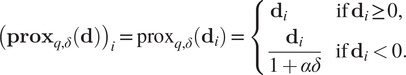


### 4.3. Intensity and background estimation results

**Figure 6.**
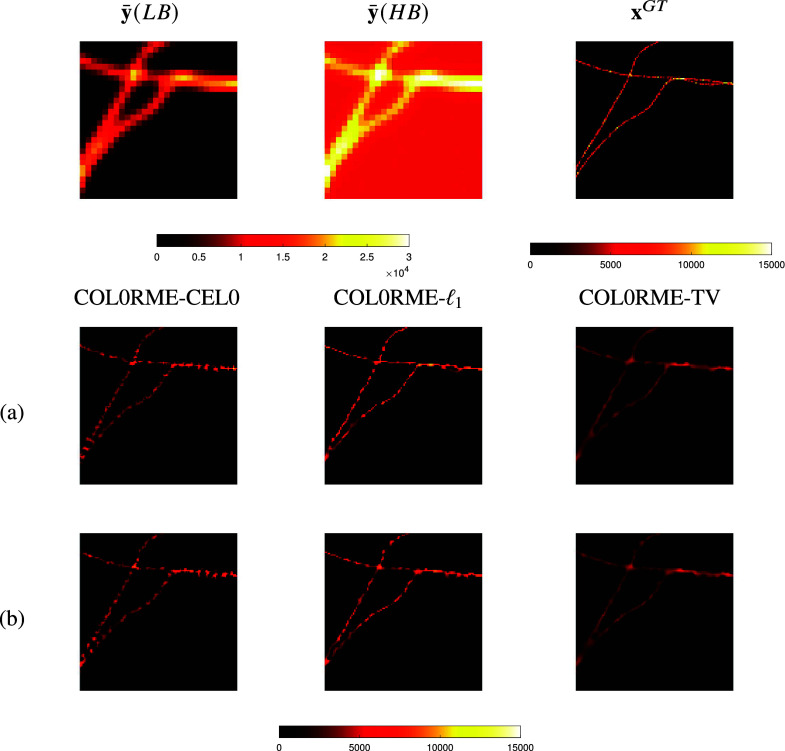
On top: Diffraction limited image 
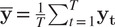
, with *T* = 500 (4× zoom) for the low-background (LB) dataset and for the high-background (HB) dataset, ground truth (GT) intensity image. (a) Reconstructions for the noisy simulated dataset with LB. (b) Reconstruction for the noisy simulated dataset with HB. From left to right: intensity estimation result on estimated support using CEL0 regularization, 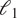 regularization and TV regularization. For all COL0RME intensity estimations, the same colorbar, presented at the bottom of the figure, has been used.

Intensity estimation results can be found in Figure 6 where (13) is used for intensity/background estimation on the supports 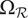 estimated from the first step of COL0RME using 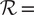
CEL0, 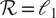, and 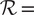
TV. We are referring to them as COL0RME-CEL0, COL0RME-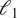, and COL0RME-TV, respectively. The colormap ranges are different for the coarse-grid and fine-grid representations, as explained in Section 6.1 The result on 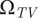, even after the second step does not allow for the observation of a few significant details (e.g., the separation of the two filament on the bottom left corner) and that is, why it will not further discussed.

**Figure 7.**
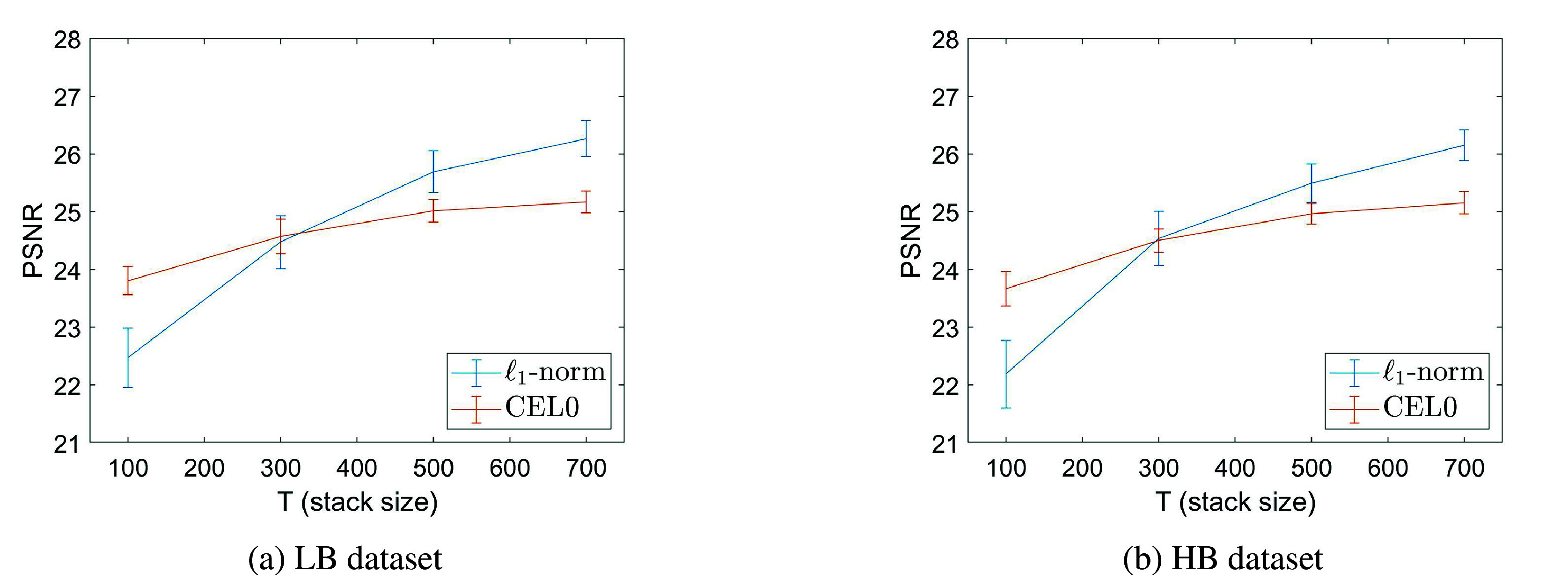
COL0RME peak-signal-to-noise-ratio (PSNR) values for two different datasets (low-background and high-background datasets), stack sizes and regularization penalty choices. The mean and the standard deviation of 20 different noise realizations are presented.

A quantitative assessment for the other two regularization penalty choices, 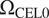 and 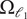, is available in Figure 7. More precisely, we compute the peak-signal-to-noise-ratio (PSNR), given the following formula:(23)
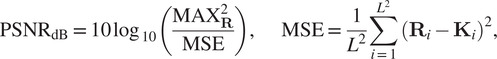
where 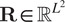 is the reference image, 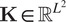 the image we want to evaluate using the PSNR metric and 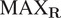 the maximum value of the image 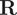. In our case, the reference image is the ground truth intensity image: 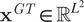. The higher the PSNR, the better the quality of the reconstructed image.

According to Figures 6 and 7, when only a few frames are considered (e.g., 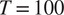 frames, high temporal resolution), the method performs better using the CEL0 penalty for the support estimation. However, when longer temporal sequences are available (e.g., 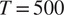 or 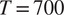 frames) the method performs better using the 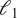-norm instead. In addition to this, for both penalizations, PSNR improves as the number of temporal frames increases.

**Figure 8.**
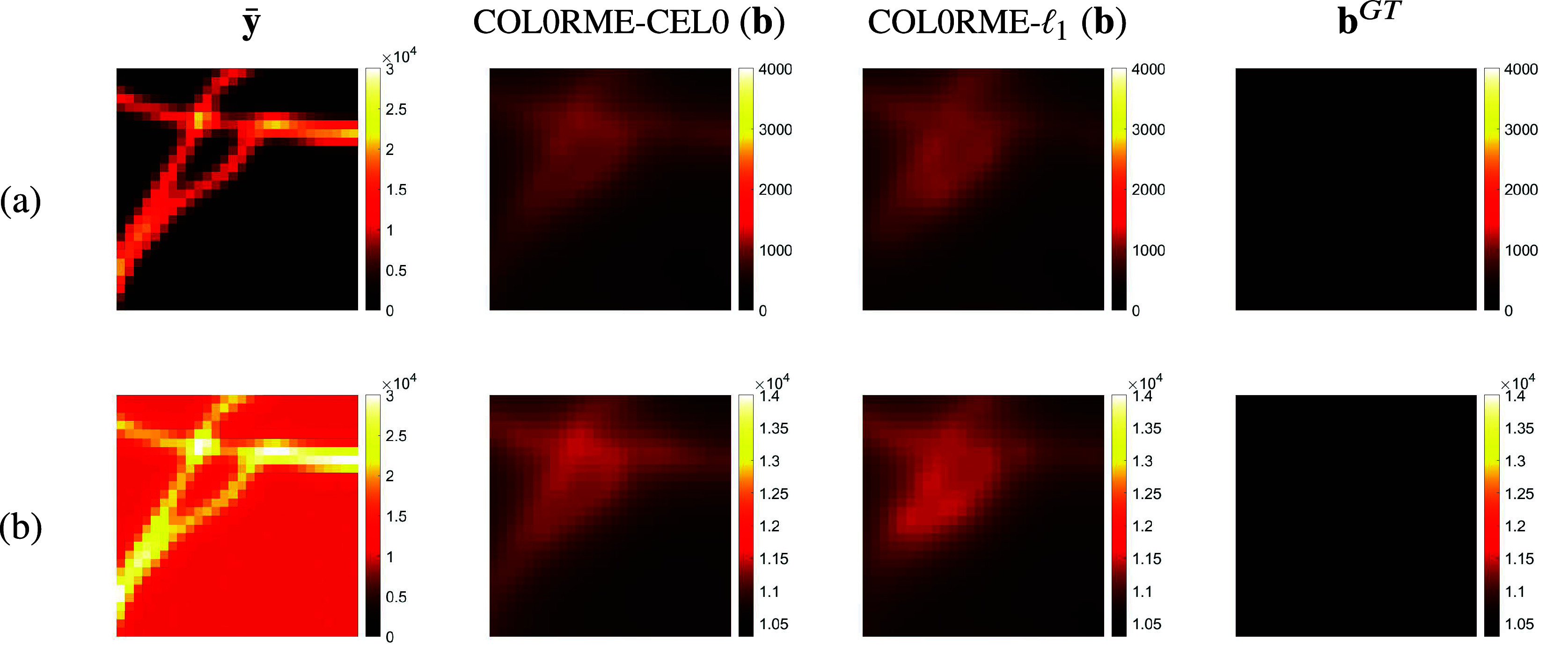
(a) Low-background (LB) dataset: Diffraction limited image 
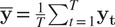
 with *T* = 500 (4× zoom), background estimation result on estimated support using CEL0 and 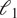 regularization, ground truth (GT) background image. (b) High-background (HB) dataset: Diffraction limited image 
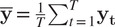
 with *T* = 500 (4× zoom), Background estimation result on estimated support using CEL0 and 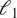 regularization, GT background image. Please note the different scales between the diffraction limited and background images for a better visualization of the results.

Background estimation results are available in Figure 8 where (13) is used for intensity/background estimation on the supports 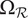, with 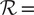 CEL0 and 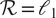, that have been already estimated in the first step. In the figure, there is also the constant background generated by the SOFI Simulation Tool^(^^31^^)^, the software we used to generate our simulated data (more details in Section 6.1). Although the results look different due to the considered space-variant regularisation on 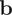, the variations are very little. The estimated background is smooth, as expected, while higher values are estimated near the simulated filaments and values closer to the true background are found away from them.

## 5. Automatic Selection of Regularization Parameters

We describe in this section two parameter selection strategies addressing the problem of estimating the regularization parameters 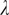 and 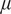 appearing in the COL0RME support estimation problem (7) and intensity estimation one (12), respectively. The other two regularization parameters 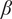 and 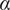 do not need fine tuning. They are both chosen arbitrary high, so as with large enough 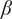 to allow for a very smooth background and with very high 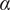 to respect the required constraints (positivity for both intensity and background and restriction to the predefined support only for the intensity estimation).

### 5.1. Estimation of support regularization parameter λ

The selection of the regularization parameter value 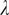 in (7) is critical, as it determines the sparsity level of the support of the emitters. For its estimation, we start by computing a reference value 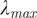, defined as the smallest regularization parameter for which the identically zero solution is found. It is indeed possible to compute such a 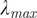 for both regularization terms CEL0 and 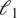 (see Refs. (32) and (33)). Once such values are known, we thus need to find a fraction 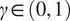 of 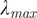 corresponding to the choice 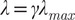. For the CEL0 regularizer the expression for 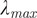 (see Proposition 10.9 in Ref. (32)) is:(24)
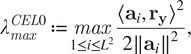
where 
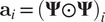
 denotes the 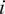
th column of the operator 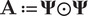. Regarding the 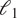-norm regularization penalty, 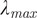 is given as follows:(25)
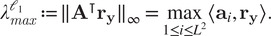


**Figure 9.**
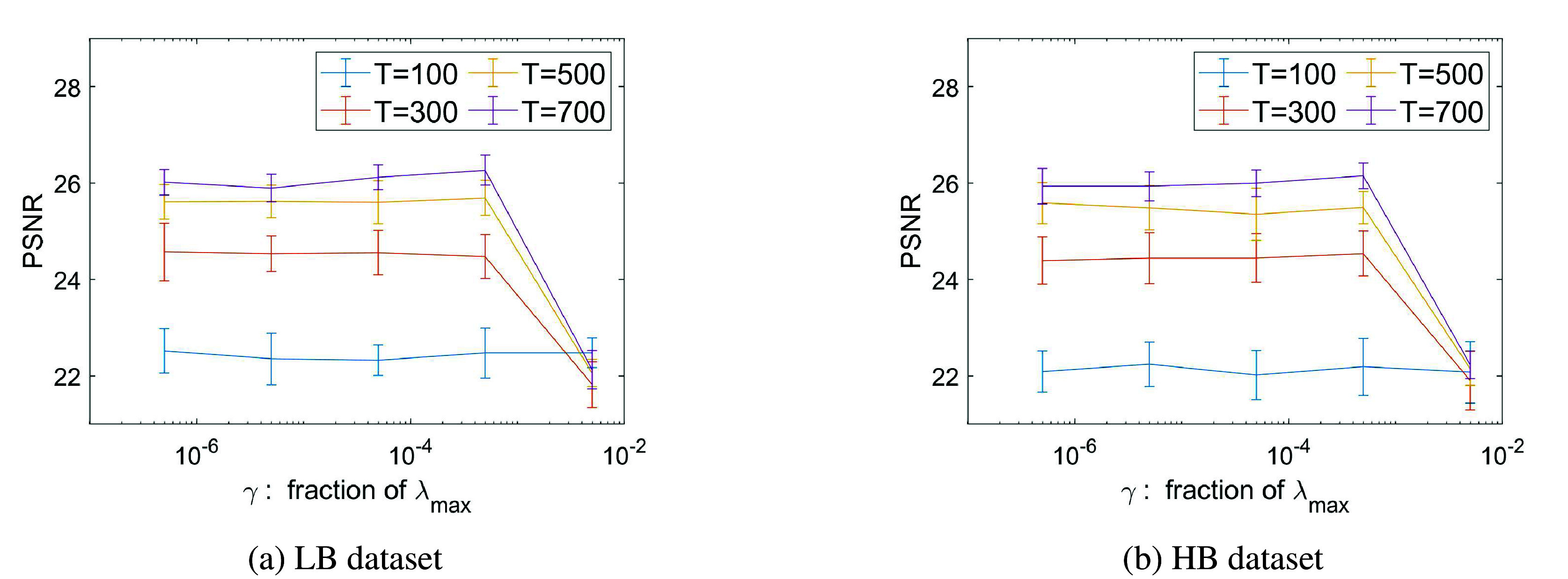
The peak-signal-to-noise-ratio (PSNR) value of the final COL0RME image, using the 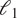-norm regularizer for support estimation, for different 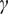 values, evaluating in both the low-background (LB) and high-background (HB) datasets. The mean and the standard deviation of 20 different noise realization are presented.

As far as 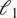 is used as regularization term in (7), we report in Figure 9 a graph showing how the PSNR value of the final estimated intensity image (i.e., after the application of the second step of COL0RME) varies for the two datasets considered depending on 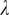. It can be observed that for a large range of values 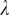, the final PSNR remains almost the same. Although this may look a bit surprising at a first sight, we remark that such a robust result is due, essentially, to the second step of the algorithm where false localizations related to an underestimation of 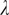 can be corrected through the intensity estimation step. Note, however, that in the case of an overestimation of 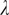, points contained in the original support are definitively lost so no benefit is obtained from the intensity estimation step, hence the overall PSNR decreases.

When the CEL0 penalty is used for support estimation, a heuristic parameter selection strategy can be used to improve the localization results, but also to avoid the fine parameter tuning. More specifically, the nonconvexity of the model can be used by considering an algorithmic restarting approach to improve the support reconstruction quality. In short, a value of 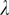 can be fixed, typically 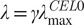 with 
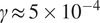
, so as to achieve a very sparse reconstruction. Then, the support estimation algorithm can be run and iteratively repeated with a new initialization (i.e., restarted) several times. While keeping 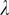 fixed along this procedure, a wise choice of the initialization depending, but not being equal to the previous output can be used to enrich the support, see Appendix C for more details. Nonconvexity is here exploited by changing, for a fixed 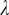, the initialization at each algorithmic restart, so that new local minimizers (corresponding to possible support points) can be computed. The final support image can thus be computed as the superposition of the different solutions computed at each restarting. In such a way, a good result for a not-finely-tuned value of 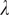 can be computed.

### Estimation of intensity regularization parameter 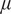 by discrepancy principle

5.2.

In this section, we provide some details on the estimation of the parameter 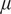 in (12), which is crucial for an accurate intensity estimation. Recall that the problem we are looking at in this second step is(26)
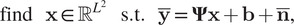
where the quantities correspond to the temporal averages of the vectorized model in (4), so that 
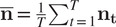
. The temporal realizations 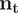 of the random vector 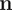 follow a normal distribution with zero mean and covariance matrix 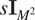, where 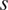 has been estimated in the first step of the algorithm, see Section 3.1. Consequently, the vector 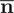 follows also a normal distribution with zero mean and covariance matrix equal to 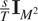. As both 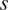 and 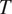 are known, we can use the discrepancy principle, a well-known *a posteriori* parameter-choice strategy (see, e.g., Refs. (34) and (35)), to efficiently estimate the hyper-parameter 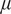. To detail how the procedure is applied to our problem, we write 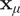 in the following to highlight the dependence of 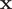 on 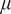. According to the discrepancy principle strategy, the regularization parameter 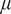 is chosen so that the residual norm of the regularized solution satisfies:(27)
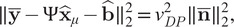
where 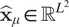 and 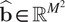 are the solutions of (12). The expected value of 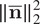 is:(28)
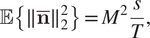
which can be used as an approximation of 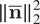 for 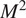 big enough. The scalar value 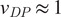 is a “safety factor” that plays an important role in the case when a good estimate of 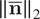 is not available. In such situations, a value 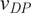 closer to 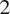 is used. As detailed in Section 3.1, the estimation of 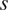 is rather precise in this case, hence we fix 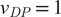 in the following.

We can now define the function 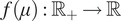 as:(29)
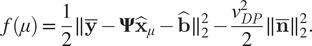
We want to find the value 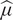 such that 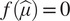. This can be done iteratively, using the Newton’s method whose iterations read:(30)
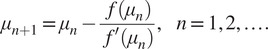


In order to be able to compute easily the values 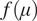 and 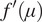, the values 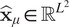, 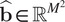, and 
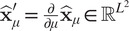
 need to be computed, as it can be easily noticed by writing the expression of 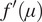 which reads:(31)
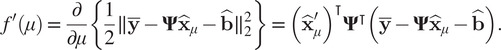


The values 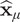 and 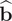 can be found by solving the minimization problem (12). As far as 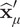 is concerned, we report in Appendix B the steps necessary for its computation. We note here, however, that in order to compute such a quantity, the relaxation of the support/non-negativity constraints by means of the smooth quadratic terms discussed above is fundamental. One can show that 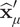 is the solution of the following minimization problem:(32)
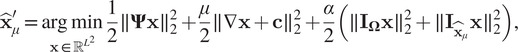
where 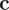 is a known quantity defined by 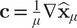, and the diagonal matrix 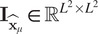 identifies the support of 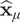 by:
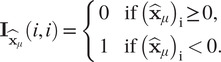


We can find 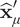 by iterating(33)
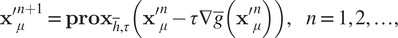
where(34)
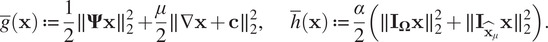


**Figure 10.**
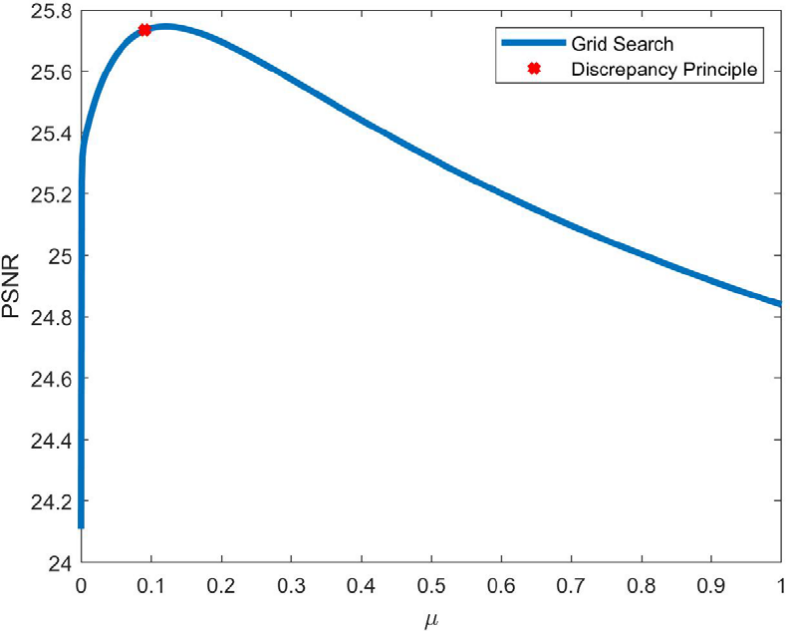
The solid blue line shows the peak-signal-to-noise-ratio (PSNR) values computed by solving (13) for several values of 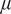 within a specific range. Tha data used are the high-background (HB) dataset with 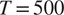 frames (Figure 12c) and the 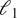-norm regularization penalty. The red cross shows the PSNR value 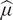 obtained by applying the Discrepancy Principle. We note that such value is very close to one maximizing the PSNR metric.

For 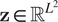, the proximal operator 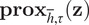 can be obtained following the computations in Appendix A:(35)
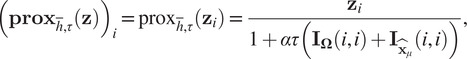
while(36)
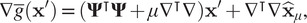
and the step 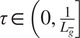, with 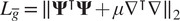 the Lipschitz constant of 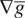. A pseudo-code explaining the procedure we follow to find the optimal 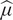 can be found in Algorithm 3. Finally, in Figure 10, a numerical example is available to show the good estimation of the parameter 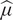.

Algorithm 3 **Discrepancy Principle.**

**Require**: 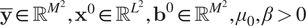,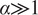.

**repeat.**

Find 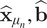 using Algorithm 2

Find 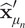 solving (32)

Compute 
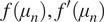
 from (29) and (31)

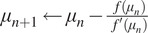

**until** convergence.

**return** 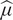.

## 6. Results

In this section, we compare the method COL0RME with state-of-the-art methods that exploit the temporal fluctuations/blinking of fluorophores, while applying them to simulated and real data. More precisely, we compare: COL0RME-CEL0 (using the CEL0 regularization in the support estimation), COL0RME-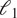 (using the 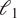-norm regularization in the support estimation), SRRF^(^^13^^)^, SPARCOM^(^^18^^)^, and LSPARCOM^(^^19^^)^. We further performed preliminary comparisons also with the ESI, 3B, and bSOFI approaches using available codes provided by the authors on the web,^1^ but we did not successfully obtain satisfactory results, so we omit them in the following.

### 6.1. Simulated data

To evaluate the method COL0RME we choose images of tubular structures that simulate standard microscope acquisitions with standard fluorescent dyes. In particular, the spatial pattern (see Figure 12a) is taken from the MT0 microtubules training dataset uploaded for the SMLM Challenge of 2016.^2^ The temporal fluctuations are obtained using the SOFI simulation tool^(^^31^^)^. This simulation software, implemented in Matlab, generates realistic stacks of images, similar to the ones obtained from real microscopes, as it makes use of parameters of the microscope setup and some of the sample’s main properties. However, differently from the fluctuating^3^ microscopic data presented in Section 6.2, the blinking generated by the SOFI Simulation Tool have a more distinctive “on–off” behavior.

**Figure 11.**
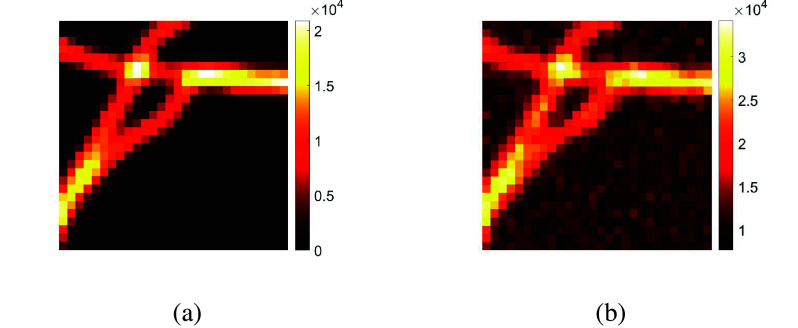
One frame of the high-background (HB) dataset, before and after the addition of background and the simulated noise degradation. (a) A convoluted and down-sampled image 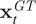 obtained from a ground truth frame 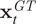, (b) a frame of the final noisy sequence: 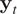. Note the different colormaps to better capture the presence of noise and background.

**Figure 12.**
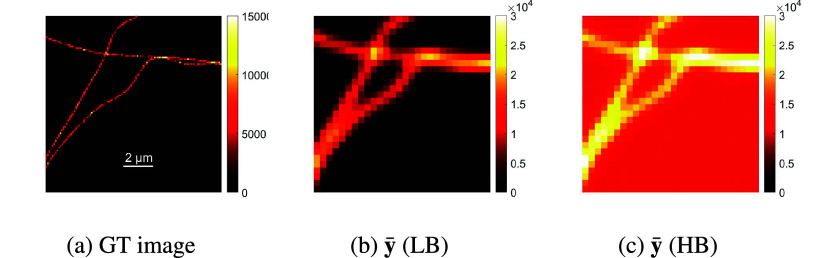
The ground truth (GT) intensity image, as well as, the diffraction limited images 
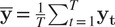
 for the two datasets with a 4× zoom, for a sequence of *T* = 500 frames.

For the experiments presented in this paper, we generate initially a video of 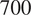 frames, however we evaluate the methods using the first 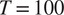, 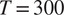, 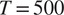, and 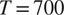 frames, so as to examine further the trade-off between temporal and spatial resolution. The frame rate is fixed at 100 frames per second (fps) and the pixel size is 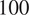 nm. Regarding the optical parameters, we set the numerical aperture equal to 1.4 and the emission wavelength to 525 nm, while the FWHM of the PSF is equal to 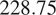 nm. The fluorophore parameters are set as follows: 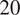 ms for on-state average lifetime, 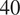 ms for off-state average lifetime and 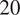 s for average time until bleaching. The emitter density is equal to 10.7 emitters/pixel/frame, while 500 photons are emitted, on average, by a single fluorescent molecule in every frame.

We create two datasets with the main difference between them being the background level, as in real scenarios the background is usually present. More precisely we create: the LB dataset, where the background is equal to 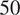 photons/pixel/frame and, the most realistic of the two, the HB dataset, where the background is equal to 2,500 photons/pixel/frame. In both datasets, we proceed as follows: initially, Poisson noise is added to simulate the photon noise (see (2)); subsequently, the number of photons recorded by each camera pixel is converted into an electric charge in accordance with the quantum efficiency and gain of the camera that have been set to 0.7 and 6, respectively (thus resulting in an overall gain of 4.2); finally, Gaussian noise is added. In order to give a visual inspection of the background and noise, in Figure 11, one frame of the HB dataset is presented before and after the background/noise addition. As we want, also, to provide a quantitative assessment, we measure the quality of the reconstruction of the final sequence of 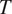 frames (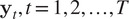) using the signal-to-noise-ration (SNR) metric, given by the following formula:(37)
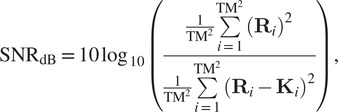
where 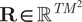 is the reference image and 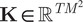 the image we want to evaluate, both of them in a vectorized form. As reference, we choose the sequence of convoluted and down-sampled ground truth frames (see one frame of the reference sequence in Figure 11a). The SNR values for a sequence of 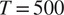 frames for the LB and HB dataset are 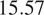 dB and 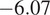 dB, respectively. A negative value is computed for the HB dataset due to the very high background used in this case.

The diffraction limited image (the average image of the stack) of each dataset as well as the ground truth intensity image are available in Figure 12. In the LB dataset, due to the high signal values, the background is not visible. Further, as the observed microscopic images and the reconstructed ones belong to different grids, coarse and fine grid respectively, their intensity values are not comparable and we can not use the same colorbar to represent them. The intensity of one pixel in the coarse grid is the summation of the intensities of 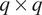 pixels in the fine grid, where 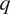 is the super-resolution factor. For this reason, we use two different colorbars.

The comparison of the method COL0RME with other state-of-the-art methods that take advantage of the blinking fluorophores is available below. Regarding the method COL0RME-CEL0 and COL0RME-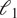, a regularization parameter equal to 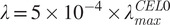 and 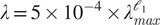, respectively, is used in the support estimation. The hyper-parameters 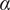 and 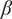 are set as follows: 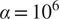, 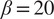. For the method COL0RME-CEL0 the algorithmic restarting approach is used for a better support estimation. It stops when there are not additional pixels added to the estimated support or if a maximum number of 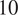 restarts is reached. Such number was empirically determined by preliminary simulations. For the method SRRF, we are using the NanoJ SRRF plugin for ImageJ.^4^ Concerning the method SPARCOM, we make use of the Matlab code available online.^5^ As regularization penalty we choose the 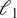-norm with a regularization parameter equal to 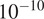 and we avoid the postprocessing step (the convolution with a small Gaussian function) for most precise localization. Finally, we test the method LSPARCOM, using the code that is, available online^6^ and the tubulin (TU) training set that is provided.

**Figure 13.**
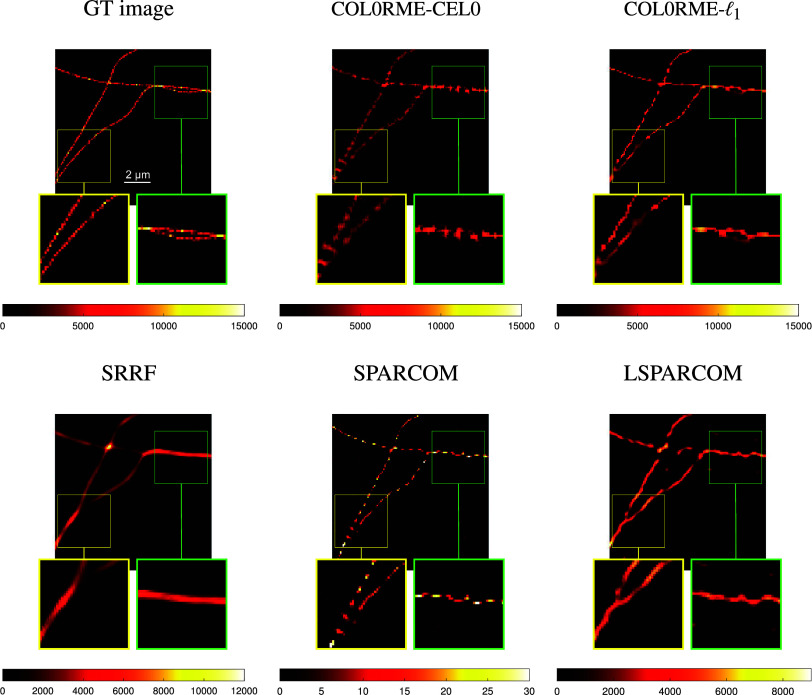
Results for the low-background (LB) dataset with 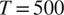. Note that the methods super-resolution radial fluctuations (SRRF), SPARCOM, and LSPARCOM do not estimate real intensity values. Between the compared methods only COL0RME is capable of estimating them, while the other methods estimate the mean of a radiality image sequence (SRRF) and normalized autocovariances (SPARCOM, LSPARCOM).

**Figure 14.**
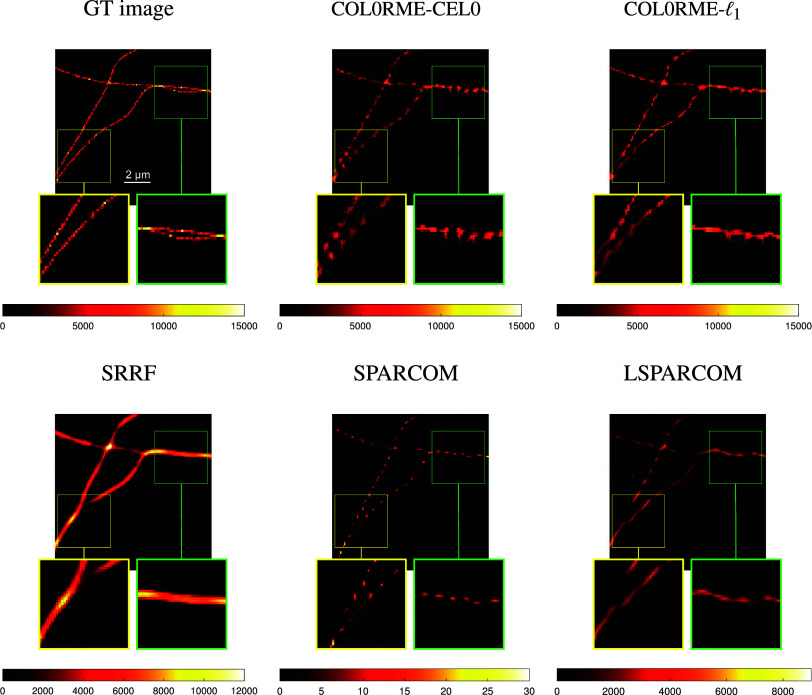
Results for the high-background (HB) dataset with 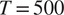.

In Figure 13, we compare the reconstructions of the methods COL0RME-CEL0, COL0RME-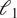, SRRF, SPARCOM, and LSPARCOM for the LB dataset and in Figure 14 for the HB dataset, for a sequence of *T* =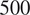 frames. Results for different stack sizes, are available in the Supplementary Figures S1–S3. Quantitative metrics like the JI for the localization precision and the PSNR for the evaluation of the estimated intensities, are only available for the methods COL0RME-CEL0 and COL0RME-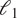 (see Figures 4 and 7). For the rest of the methods, the JI values are very small due to background and noise artifacts in the reconstructions that lead to the appearance of many false positives, while the PSNR metric is not possible to be computed as the methods SRRF, SPARCOM, and LSPARCOM do not reconstruct the intensity level. In both datasets, LB and HB datasets, and for a sequence of *T* = 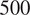 frames, the better reconstruction, visually, is the one of the method COL0RME-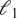, as it is able to achieve a more clear separation of the filaments in the critical regions (yellow and green zoom boxes). The method COL0RME-CEL0 achieves also a good result, eventhough the separation of the filaments, that are magnified in the green box, is not so obvious. The same happens also when the method SPARCOM is being used. Finally, the reconstruction of the methods SRRF and LSPARCOM, is slightly misleading.

### 6.2. Real data

To show the effectiveness of our method for handling real-world data, we apply COL0RME to an image sequence acquired from a TIRF microscope. The TIRF microscope offers a good observation of the activities happening next to the cell membrane, as it uses an evanescent wave to illuminate and excite fluorescent molecules only in this restricted region of the specimen^(^^36^^)^. Further, the TIRF microscope does not require specific fluorescent dyes, allows live cell imaging using a low illumination laser, with really low out-of-focus contribution and produces images with a relatively good, in comparison with other fluorescence microscopy techniques, SNR. To enhance the resolution of the images acquired from a TIRF microscope, super-resolution approaches that exploit the temporal fluctuations of blinking/fluctuating fluorophores, like COL0RME, can be applied.

**Figure 15.**
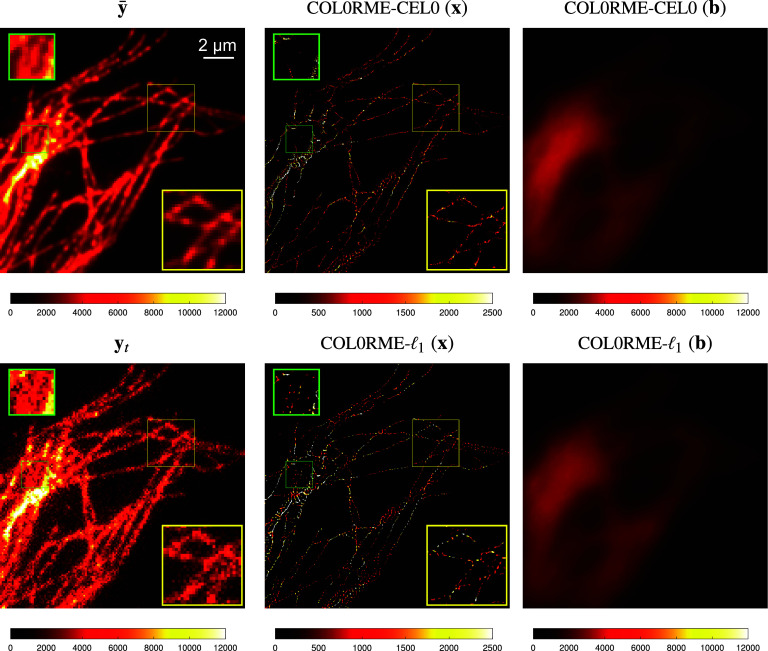
Real total internal reflection fluorescence (TIRF) data, 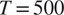 frames. Diffraction limited image or the mean of the stack 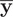 (4× zoom), a frame 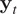 from the stack (4× zoom), the intensity and background estimation of the methods COL0RME-CEL0 and COL0RME-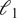.

The data we are using have been obtained from a multiangle TIRF microscope, with a fixed angle close to the critical one. A sequence of 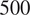 frames has been acquired, with an acquisition time equal to 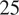 s. Tubulins in endothelial cells are being imaged, while they are colored with the Alexa Fluor 488. The variance of fluctuations over time for a typical pixel is measured and is belonging to the range 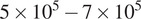. The diffraction limited image, or with other words the mean stack image 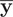 is shown in Figure 15, together with one frame 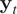 extracted from the entire stack. The FWHM of the PSF has been measured experimentally and is equal to 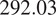 nm, while the CCD camera has a pixel of size 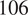 nm.

The results of the method COL0RME-CEL0 and COL0RME-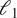 and more precisely the intensity and the background estimation, can be found in Figure 15. Experiments using different stack sizes have been done showing that the more frames we use (up to a point that we do not have many molecules bleached), the more continuous filaments we find. However, by acquiring only 500 frames we have a good balance between temporal and spatial resolution. For this reason we present here only results using a stack of 500 frames. For the method COL0RME-CEL0 the regularization parameter 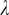 is equal to 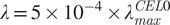 and the algorithmic restarting approach has been used (stopping criteria: when, in a certain restarting, there are not additional pixels added to the global support, but with maximum 10 restarts). Regarding the method COL0RME-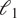 the regularization parameter 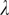 is equal to 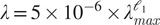, a relatively small value so as to be sure that we will include all the pixels that contain fluorescent molecules. Even if we underestimate 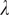 and find more false positives in the support estimation, after the second step of the algorithm, the final reconstruction is corrected, as explained in 5.1. The hyper-parameters 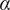 and 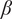 are equal to: 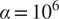, 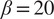. Using any of the two regularizers the spatial resolution is enhanced, as it can be also observed from the yellow zoom boxes. However, the reconstruction obtained by both COL0RME-CEL0 and COL0RME-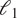 is to some degree punctuated due to mainly limitations arising from experimental difficulties to get a staining sufficiently homogeneous for this imaging resolution. Furthermore, there are a few filaments that do not seem to be well reconstructed, especially using the COL0RME-CEL0 method, for example, the one inside the green box.

**Figure 16.**
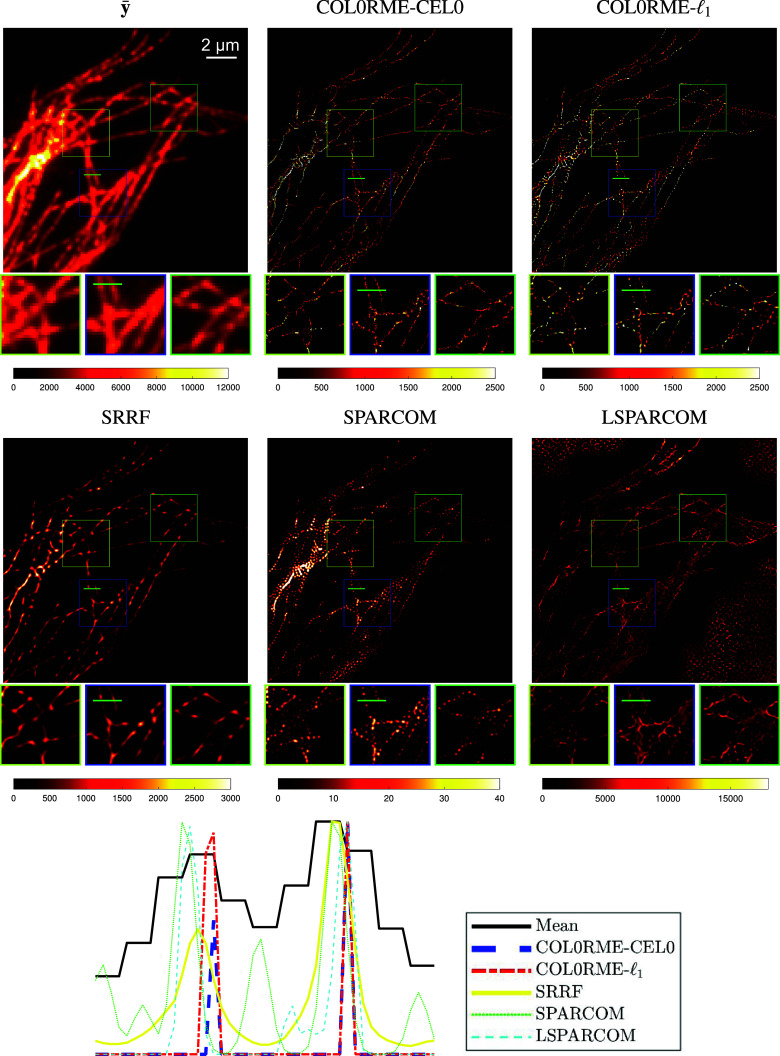
Real total internal reflection fluorescence (TIRF) data, 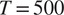 frames. Diffraction limited image 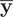 (4× zoom), comparisons between the method that exploit the temporal fluctuations, normalized cross-section along the green line presented in the diffraction limited and reconstructed images, but also in the blue zoom-boxes. Discription of colorbars: real intensity values for 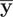 and COL0RME in two different grids, mean of the radiality image sequence for super-resolution radial fluctuations (SRRF), normalized autocovariances for SPARCOM and LSPARCOM.

Finally, the comparison of the methods COL0RME-CEL0 and COL0RME-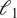 with the other state-of-the-art methods is available in Figure 16. The parameters used for the methods SRRF, SPARCOM, and LSPARCOM, are explained in the Section 6.1. Here, we further use the postprocessing step (convolution with a small Gaussian function) in the method SPARCOM, as the result was dotted. The methods COL0RME-CEL0 and COL0RME-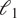 seem to have the most precise localization, by reconstructing thin filaments, as shown in the cross-section plotted in Figure 16, although a bit punctuated. The most appealing visually is the result of the method SRRF, where the filaments have a more continuous structure, however from the cross-section, we can see that the resolution is not so much improved compared to the other methods. SPARCOM and LSPARCOM do not perform very well in this real image sequence due to, mainly, background artifacts.

**Figure 17.**
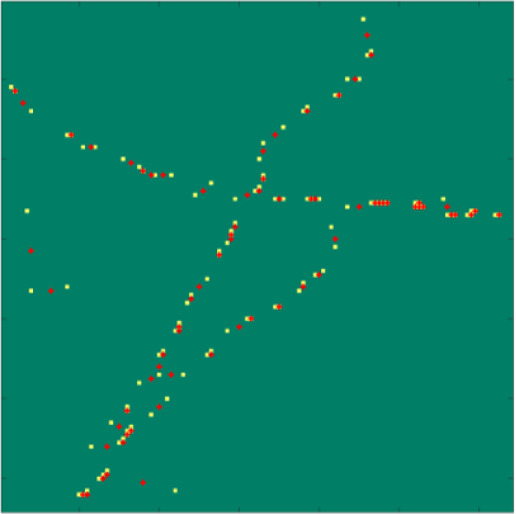
The yellow pixels belong to the support estimated in the previous restarting, while the red pixels belong to the initialization that is, used in the current restarting.

## 7. Discussion and Conclusions

In this paper, we propose and discuss the model and the performance of COL0RME, a method for super-resolution microscopy imaging based on the sparse analysis of the stochastic fluctuations of molecules’ intensities. Similarly to other methods exploiting temporal fluctuations, COL0RME relaxes all the requirements for special equipment (microscope and fluorescent dyes) and allows for live-cell imaging, due to the good temporal resolution and the low power lasers employed. In comparison with competing methods, COL0RME achieves higher spatial resolution than other methods exploiting fluctuations while having a sufficient temporal resolution. COL0RME is based on two different steps: a former one where accurate molecule localization and noise estimation are achieved by solving nonsmooth convex/nonconvex optimization problems in the covariance domain and the latter where intensity information is retrieved in correspondence with the estimated support only. Our numerical results show that COL0RME outperforms competing approaches in terms of localization precision. To the best of our knowledge, COL0RME is the only super-resolution method exploiting temporal fluctuations which is capable of retrieving intensity-type information, signal and spatially varying background, which are of fundamental interest in biological data analysis. For both steps, automatic parameter selection strategies are detailed. Let us remark that such strategy of intensity estimation could be applied to the other competing super-resolution methods in the literature. Several results obtained on both simulated and real data are discussed, showing the superior performance of COL0RME in comparison with analogous methods such as SPARCOM, LSPARCOM and SRRF. Possible extensions of this work shall address the use of intensity information estimated by COL0RME for 3D reconstruction in, for example, MA-TIRF acquisitions. Furthermore, a systematic study to assess quantitatively the spatial resolution achieved by COL0RME under different scenarios (different background levels, different PSNRs, and number of frames) is envisaged.

## Data Availability

Replication data and code can be found in: https://github.com/VStergiop/COL0RME.

## Footnotes

1 ESI: https://github.com/biophotonics-bielefeld/ESI, 3B: http://www.coxphysics.com/3b, bSOFI implemented in SOFI Simulation Tool software package: https://github.com/lob-epfl/sofitool.

2 http://bigwww.epfl.ch/smlm/datasets/index.html.

3 The emission of a single fluorophore over time can be described by a Poisson distribution.

4 https://github.com/HenriquesLab/NanoJ-SRRFhttps://github.com/HenriquesLab/NanoJ-SRRF.

5 https://github.com/KrakenLeaf/SPARCOMhttps://github.com/KrakenLeaf/SPARCOM.

6 https://github.com/gilidar/LSPARCOMhttps://github.com/gilidar/LSPARCOM.

## Supplementary material

For supplementary material accompanying this paper visit https://doi.org/10.1017/S2633903X22000010. click here to view supplementary material

## A. Appendix. Proximal Computations

Given the function , defined in (17), the proximal mapping of  is a an operator given by:

(A.1)

The optimal solution  (), as the problem (A.1) is convex, is attained when:

(A.2)

Starting from (14), we can compute , as:

(A.3)

Given (A.3), we can write:

(A.4)

Exploiting component-wise, as problem (A.1) is separable with respect to both  and , and assuming , the derivative computed at (A.4) vanishes for:

(A.5)

and it holds for . Similarly, for the case , this analysis yields:

(A.6)

for .

So finally, the proximal operator is given by:

(A.7)

In a similar way, we compute the proximal mapping of the function , defined in (34), as follows:

(A.8)

The optimal solution  of (A.8)  is attained when:

_(A.9)_

By eliminating  in the expression (_A.9_), we compute element-wise the proximal operator:

(A.10)

## B. Appendix. The Minimization Problem to Estimate

Starting from the penalized optimization problem (13) and having  fixed, we aim to find a relation that contains the optimal . While there are only quadratic terms, we proceed as following:

(A.11)

Given (A.3), we can write:

(A.12)

Our goal is to compute , the partial derivative of  w.r.t. . So, we derive as follows:

_(A.13)_

We define the matrix  such as:

Now the vector , using further the equation (A.3), can be simply written as:  and then (_A.13_) becomes:

The minimization problem we should solve in order to find  thus is:

(A.14)

## C. Appendix. Algorithmic Restart

Every initialization is based on the solution obtained at the previous restarting. There are many ways to choose the new initialization, deterministic, and stochastic ones. In this paper, we chose a deterministic way based on the following idea: for every pixel belonging to the solution of the previous restarting we find its closest neighbor. Then, we define the middle point between the two and we include it in the initialization of the current restarting. A small example is given in the Figure 17. The yellow points belong to the support estimation of the previous restarting. Starting from them we define the red points, used for the initialization of the current restarting.
